# Bright Field Microscopy as an Alternative to Whole Cell Fluorescence in Automated Analysis of Macrophage Images

**DOI:** 10.1371/journal.pone.0007497

**Published:** 2009-10-22

**Authors:** Jyrki Selinummi, Pekka Ruusuvuori, Irina Podolsky, Adrian Ozinsky, Elizabeth Gold, Olli Yli-Harja, Alan Aderem, Ilya Shmulevich

**Affiliations:** 1 Institute for Systems Biology, Seattle, Washington, United States of America; 2 Department of Signal Processing, Tampere University of Technology, Tampere, Finland; 3 Department of Bioengineering, University of Washington, Seattle, Washington, United States of America; 4 Department of Electrical Engineering, University of Washington, Seattle, Washington, United States of America; National Microelectronics Center, Spain

## Abstract

**Background:**

Fluorescence microscopy is the standard tool for detection and analysis of cellular phenomena. This technique, however, has a number of drawbacks such as the limited number of available fluorescent channels in microscopes, overlapping excitation and emission spectra of the stains, and phototoxicity.

**Methodology:**

We here present and validate a method to automatically detect cell population outlines directly from bright field images. By imaging samples with several focus levels forming a bright field 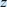 -stack, and by measuring the intensity variations of this stack over the 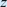 -dimension, we construct a new two dimensional projection image of increased contrast. With additional information for locations of each cell, such as stained nuclei, this bright field projection image can be used instead of whole cell fluorescence to locate borders of individual cells, separating touching cells, and enabling single cell analysis. Using the popular CellProfiler freeware cell image analysis software mainly targeted for fluorescence microscopy, we validate our method by automatically segmenting low contrast and rather complex shaped murine macrophage cells.

**Significance:**

The proposed approach frees up a fluorescence channel, which can be used for subcellular studies. It also facilitates cell shape measurement in experiments where whole cell fluorescent staining is either not available, or is dependent on a particular experimental condition. We show that whole cell area detection results using our projected bright field images match closely to the standard approach where cell areas are localized using fluorescence, and conclude that the high contrast bright field projection image can directly replace one fluorescent channel in whole cell quantification. Matlab code for calculating the projections can be downloaded from the supplementary site: http://sites.google.com/site/brightfieldorstaining

## Introduction

The development of highly specific stains and probes, for example the green fluorescent protein and its derivatives, have made fluorescence microscopy the standard tool for visualization and analysis of cellular functions and phenomena. On the other hand, automated microscopes and advances in digital image analysis have enabled high-throughput studies automating the imaging procedure and cell based measurements. In fluorescence microscopy of eukaryotic cells, automated single-cell quantification can be achieved using multiple fluorescent probes and channels in a single experiment. The first fluorescence channel enables detection of stained nuclei, resulting in markers for cell locations. The second fluorescent channel visualizes the areas occupied by whole cells or cytoplasm, for example by a cytoskeletal actin stain [1]. Alternatively, a nonspecific subcellular stain can be used for whole cell detection, with most fluorescence molecules located in the compartments the stain targets, but with stain residue visible in the cytoplasmic area. Regardless of the approach for whole cell staining, cells that are touching or partly overlapping can be automatically separated with the help of the nuclei markers of the first channel [2]. Finally, subcellular phenomena are quantified by measuring different properties of the first and second channels, or by using additional organelle and molecule specific probes and extra fluorescence channels, for example in colocalization measurements [3].

Because of the limited number of fluorescent channels available, and because of partly overlapping excitation and emission spectra of the probes, studies involving subcellular colocalization are commonly carried out without nuclear or whole cell staining. As a consequence, cell-by-cell measurements are not possible. Single cell measurements are also difficult or even impossible in cells that are used for negative control, where the lack of fluorescence is used for the detection of some phenomena. Furthermore, there are other limitations in fluorescence microscopy, such as phototoxicity and imaging setup complexity. These problems have motivated the search for alternate methods to replace at least some of the fluorescence channels with standard transmitted light microscopy.

The bright field channel, although readily available in all microscopes, is often neglected in cell population studies. Firstly, the cells are often nearly transparent, making the contrast very poor. Even by manual visual cell analysis it is often impossible to reliably detect the locations of cell borders, especially if the cells are clumped together. Furthermore, since no specific staining is applied, subcellular phenomena cannot be detected and nuclei are often only faintly visible. Recently, however, a number of studies have been published showing the usefulness of the bright field channel in cell detection and automated image analysis of cell populations. In Quantitative Phase Microscopy, a phase map of samples is estimated from bright field images of different focus levels [4] using proprietary software to greatly increase the contrast. In [5] a similar approach was taken, but the phase map was measured using lowpass digital filtering, followed by a computationally expensive level set based segmentation of individual cells. Texture analysis methods have also been used for bright field cell detection, such as the method presented by [6], where cell contours were extracted after initial segmentation. For round cells with rather good contrast borders, such as yeast, there are multiple algorithms available [7]–[9]. In cell tracking, the bright field cell segmentation is often presented as a preprocessing step followed by the actual tracking algorithm [10]. Utilizing bright field images with rather good contrast, it has also been shown that it is possible to classify between different cell types without fluorescent stains [11]. Finally, special microscopy techniques such as digital holography [12] have been used instead of fluorescent staining.

We introduce and validate 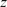 -projection based methods for replacing whole cell fluorescent staining with bright field microscopy. In the presented approaches the cells are imaged with several different focal planes as in [5] and [4], but instead of solving for the phase map, we measure the intensity variations in the 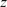 -dimension of bright field stack, creating a new 2-D image for analysis. The pixel intensities inside the cells vary when the focus is changing, but the background intensity stays more constant throughout the stack, resulting in relatively high variation inside the cells, but almost zero outside. Therefore, in the resulting projections the cells appear as brighter objects on an essentially black background, enabling us to replace the fluorescence image of whole cell staining with this bright field projection. In comparison to the previous bright field based cell segmentation techniques presented in the literature, this approach is more straightforward to implement, and the resulting bright field projection image is directly applicable for segmentation using CellProfiler [2] analysis software designed for fluorescent microscopy. Furthermore, with the exception of a preprocessing step with image filtering, no parameters need to be set when calculating the projection. As validation, we apply the technique for segmentation of mouse bone marrow derived macrophage cells with complex shapes and very low contrast. Phase contrast and differential interference contrast (DIC) microscopy techniques offer contrast increase through special optics, but to the best of our knowledge there is no work in the literature suggesting that standard cell segmentation algorithms for fluorescence microscopy would be applicable for phase or DIC images, or that the robust segmentation of cells with irregular shapes would be possible for large sets of images.

The resulting projections are shown to enable whole cell segmentation if only nuclear staining or other marker, such as manual cell marking for each cell is available, removing the need for an additional fluorescent channel for whole cell detection.

## Methods

To evaluate the performance of projection based methods, we acquired test image data by culturing and imaging bone marrow macrophages (BMM). The macrophages isolated from BL6 were cultured on glass cover slip in RPMI medium, supplemented with 10% fetal bovine serum, 100 u/ml penicillin, 100 ug/ml streptomycin, 2 mM GlutaMAX and 50 ng/ml m-CSF (37 C, 5% CO2). The cells were stimulated with LPS 100 ng/ml for 1, 2, 4, 6, 18, and 24 hours, fixed with 3% Paraformaldehyde for 20 min and stained with BODIPY 493/503 (Invitrogen) for lipid bodies, and Sytox (Invitrogen) for nuclei. Unstimulated macrophages as well as the stimulated cells of different time points were imaged with Leica DMIRB confocal laser scanning microscope.

The image stacks form eight groups with varying cell morphologies: two image sets of unstimulated macrophage cells, and a time series experiment with six groups of macrophage images from different time points during the stimulation. For each group, there are five image stacks, each consisting of three channels: 1. fluorescent nuclei 2. fluorescence subcellular stain for lipid bodies also visualizing the cytoplasm and 3. bright field channel. Each of the stacks for every channel consist of 20 individual 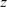 -slices. One stack for each channel of the time point 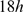 had to be removed because it was erroneously imaged as a single slice instead of a stack. In total, the test data set includes nearly 800 cells.

To enable whole cell segmentation from bright field images, the contrast must be enhanced by increasing the intensity differences between cell and background areas. We achieve this by calculating different measures of variation in the 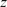 -direction, projecting the bright field stacks into two dimensional (2-D) images. That is, each pixel in the resulting 2-D projections corresponds to a measure of intensity variation in the 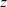 -direction in the original stack in that specific 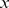, 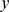 pixel location. Since there is typically less 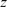 intensity variation in the background than in cells, these two classes of pixels can be separated. Specifically, we make the projections using standard deviation (STD), interquartile range (IQR), coefficient of variation (CV), and median absolute deviation (MAD) measures.

The STD projection image is constructed by calculating the standard deviation of intensities in the 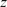 -direction for each pixel of the original stack:
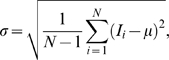
(1)where 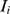 is the pixel intensity of 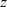 -slice 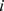, 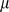 is the mean of the pixel intensities, and 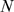 is the total number of 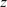 -slices.

For a more robust measure of variation we calculated IQR projection, the difference between the 75th and the 25th percentiles of the sample. That is, the lowest 25% and highest 25% of the values are first discarded, and the IQR is the range between the maximum and minimum of all the remaining intensities of 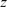 -slices.

In CV projection, the standard deviation of the 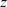 -values is divided by the mean of the values
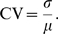
(2)


And finally, MAD measures how much “on average” one value deviates from the median of all the values, that is, the median deviation from the median of the intensities of all the 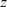 -slices for every 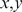 pixel location:
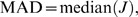
(3)where 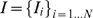, 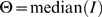, and 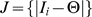.

To assess the projections' sensitivity to the number of 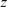 -slices imaged for each stack, we applied the STD projection to two different types of reduced stacks, consisting only of three slices. First, the three slices were selected by hand representing nearly the whole 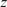 -range of the original stack (slices 2, 10 and 19), referred to as the 3Slices-method. And second, we created five reduced versions of the original stacks by selecting the three slices randomly, referred to as 3SlicesRandom1 to 3SlicesRandom5.

The automated image analysis and cell segmentation for the evaluation of the various projection methods was carried out by the open source CellProfiler software package [2], originally designed for fluorescence microscopy. First, markers for each cell were obtained by detecting fluorescent nuclei with IdentifyPrimAutomatic analysis module. Second, to smooth out small unwanted details from the projections, a Gaussian lowpass filter radius of 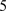 pixels was applied by SmoothOrEnhance module. Third, we used the propagation algorithm [13] in the IdentifySecondaryAutomatic module for detecting the whole cell areas. For ground truth, the whole cell areas were segmented with the same procedure (excluding the lowpass filter) using fluorescent cytoplasm images to be compared against cell area detection using the various 2-D projections. To simulate a situation where no fluorescent staining is available, the cytoplasmic areas were estimated by an annulus of radius 30 pixels around each nuclei as described, for example, in [14]. This estimation approach is referred to as the Annulus-method.

For further validation, we also enumerated fluorescent spots visible in the second fluorescent channel of the stacks. The spot enumeration was done with a kernel density estimation based algorithm [15] using a Gaussian kernel. Since this spot enumeration module is not included in the standard CellProfiler distribution, we implemented the analysis pipeline in the Developer's Version of CellProfiler, running on Matlab 2008a. The various approaches for whole cell segmentation are summarized in Table 1.

**Table 1:** Summary of different whole cell segmentation methods and abbreviations.

Description of whole cell segmentation method	Abbreviation
Standard deviation projection	STD
Interquartile range projection	IQR
Coefficient of variation projection	CV
Median absolute deviation projection	MAD
Standard deviation projection for a reduced 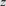 -stack with three 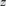 -slices (2, 10 and 19) out of the 20 in original stacks.	3Slices
Standard deviation projection for a reduced 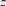 -stack with three randomly selected 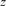 -slices. Five separate samples.	3SlicesRandom1-5
Whole cell area estimated to extend 30 pixels around the nucleus.	Annulus
Ground truth segmentation using fluorescent cytoplasm staining.	Fluorescence

Descriptions and abbreviations of all the different methods used for whole cell segmentation.

We did not discard cells touching image borders, although it is a procedure commonly performed to minimize bias in measurements caused by cells that are only partly visible. These cells allows us to compare segmentation accuracy also on image borders where image quality is often compromised due to nonuniform background. The computational complexity of the analysis is relatively low, taking around 4 seconds per method to calculate the projection and segment the image on a 2GHz PC with Windows Vista.

## Results

**Figure 1:**
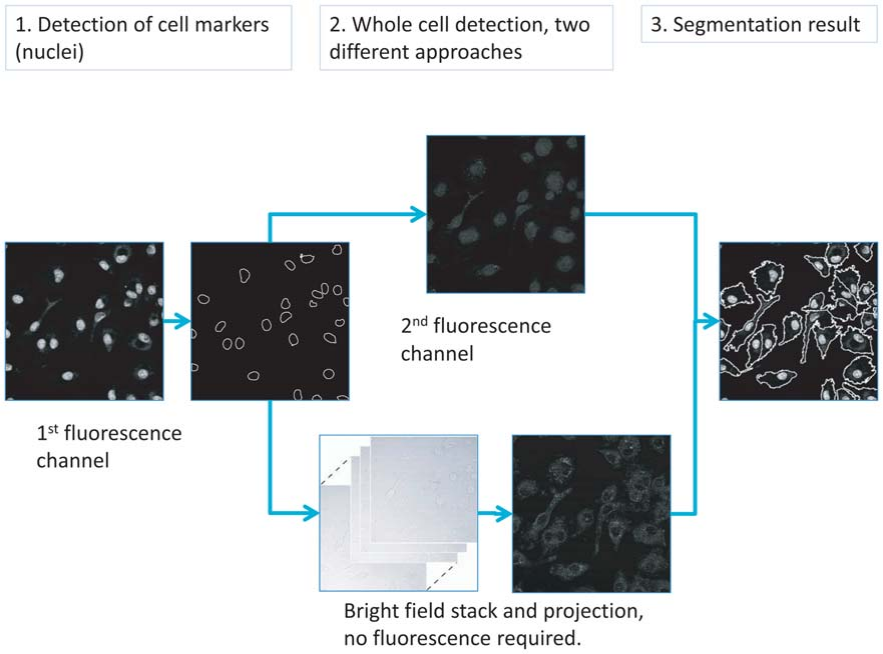
Flowchart of the cell segmentation procedure. Whole cell fluorescent staining is replaced by projection images calculated from bright field image stacks of different focal planes.

**Figure 2:**
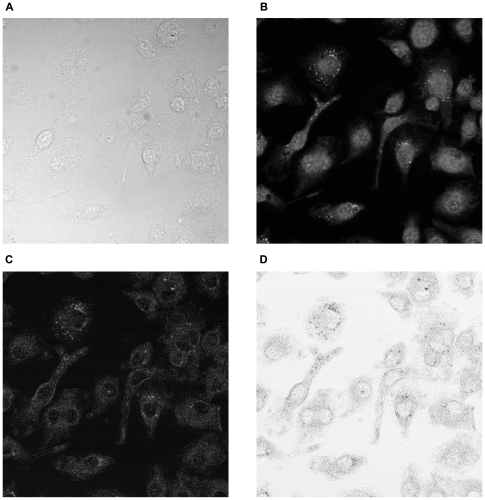
Contrast enhancement by standard deviation projection of bright field image stack. (A) Low contrast bright field image. (B) Fluorescence staining for whole cell and bright spot detection. (C) Standard deviation projection of stack of bright field images. (D) Inverse of the projection for another visualization of the projection result. In addition to increased contrast, the projection also suppresses background nonuniformities.

As described in the previous section, we projected stacks of bright field images into 2-D by various measures of stack 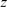 -variation, with the aim of replacing whole cell fluorescent staining. This procedure is outlined in Fig. 1, where markers for each cell are detected from fluorescence, or marked by hand, with two alternative methods for whole cell detection: fluorescence and the projections. Fig. 2 illustrates the contrast improvement by one of the projection approaches (STD). Fig. 2A shows one slice of the original bright field image, while fluorescence staining, the proposed STD projection, and the inverse of the projection are presented in Fig. 2B, 2C and 2D, respectively. The difference in contrast between the projection 2C and original bright field data 2A is easily noticeable, and furthermore, since the deviation in background intensities is similar in all the 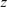 -slices, the nonuniform background is efficiently removed by the projection. The projections by all the methods for all the stacks are given in the supplementary www-pages at http://sites.google.com/site/brightfieldorstaining

**Figure 3:**
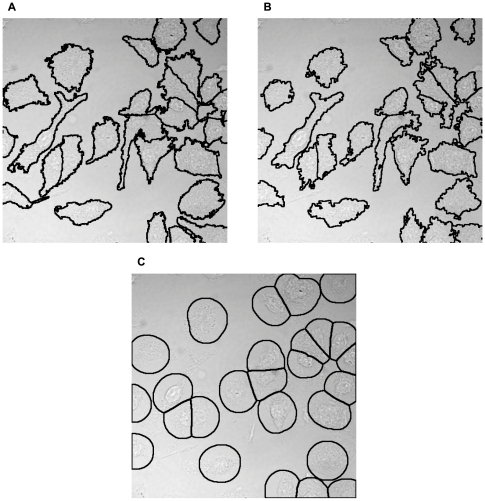
Whole cell segmentation using different input data. (A) Fluorescent whole cell staining. (B) Standard deviation projection of bright field stack. (C) The Annulus-method. The segmentation was performed using CellProfiler software, all methods requiring the use of fluorescent nuclei as markers for each cell.

For assessing the performance of the projection method, we compared automated image segmentation of whole cell areas of fluorescently stained cells to the bright field projections, and to the Annulus-method where the cytoplasm areas were estimated by annuli around the detected nuclei. We were unable to detect the cells of our whole dataset using the best previously published method in the literature for segmenting complex cell shapes in bright field images [5], and we therefore had to leave it out of this comparison study. Fig. 3 illustrates one segmentation comparison, after image analysis by CellProfiler software. Fig. 3A presents the the whole cell segmentation result using fluorescence (Fig. 2B), and in Fig. 3B the whole cell areas were detected from the projected bright field stack (Fig. 2C). Fig. 3C shows the annuli around nuclei, resulting from the Annulus method. All the methods use fluorescent nuclei as markers for each cell, around which the whole cell areas are located.

To quantify the segmentation accuracy for all the image stacks of the time series experiment, we measured the precision
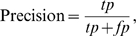
(4)and recall
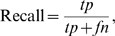
(5)where 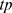, 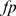, and 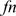 are the numbers of detected true positive, false positive, and false negative pixels, respectively [16]. Perfect precision would indicate that all the pixels detected by the method under testing (different bright field projections) are also present in the ground truth segmentation result (fluorescence). Perfect recall, on the other hand, would indicate that that no pixels of the fluorescence image are missed by using the bright field projection image.

For a more compact representation of the segmentation accuracy we computed the F-score [16]:
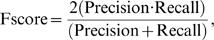
(6)that is, the harmonic mean of precision and recall. An F-score of 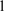 corresponds to perfect segmentation accuracy.

**Figure 4:**
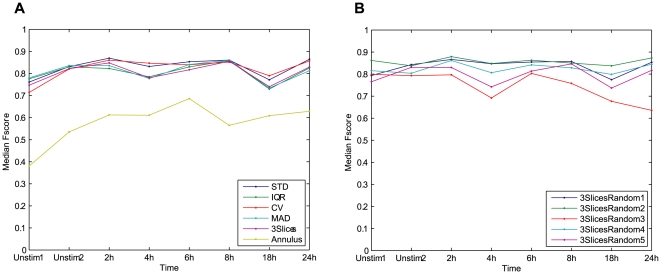
Pixel-by-pixel comparison of whole cell segmentation using bright field projections against fluorescence ground truth. (A) Median F-scores over all cells for each image group, with all the projection methods. (B) Median F-scores for cell segmentation using standard deviation projection images, each projected from three randomly selected slices.

Fig. 4A presents the per cell segmentation F-score medians over all cells for all the different projection methods against the fluorescence ground truth. Furthermore, the segmentation results for the STD projection of the 3Slices set with only 3 hand picked 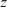 -slices are given, as well as the F-score for the Annulus method. Fig. 4B gives the segmentation results of STD projection for 3SlicesRandom1 to 5, assessing the effect of random 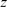 -slice selection from the stack for the projection.

With our data set consisting of nearly 800 macrophage cells with highly complex morphologies, the overall performance of the projection methods were close to the ground truth fluorescence staining with the median F-score fluctuating around 0.8. As expected, the F-score is consistently lower for the Annulus method. More extensive plots, including F-score boxplots for each method, are given at the supplementary site. The supplementary boxplots show a number of outliers for each of the of the eight groups, for all the projection methods. In comparison to the whole dataset, the number of outliers is limited, and the effect of these outliers can be reduced, for example, by discarding the corresponding cells from further analysis, similarly as cells that are too clumped together often need to be removed from automated segmentation results. As seen from the segmentation result images (supplement) the outliers were caused by segmentation errors overestimating the whole cell areas, suggesting the area of the cell to be a suitable feature for discarding these outliers if necessary.

To evaluate whether the outliers and other variations in the cell segmentation results affect the biological conclusions drawn from the data, we compared subcellular spot counts on a single cell level. By utilizing the second fluorescent channel where lipid bodies are emphasized as bright spots, we first detected the spots in the images (spot detection results for all images available in the supplement site). Then, based on the whole cell segmentation by all the projection approaches, we determined the cell to which each spot belongs. Finally, we discarded the spots outside the detected cells. This procedure enables us to estimate the effect of the different whole cell detection methods on the actual biological conclusions (spot counts per cell), since if the whole cell area detection differs dramatically from the fluorescence ground truth cell area, the numbers of spots detected in these erroneously segmented cells also change. If there is no change in spot counts, the whole cell detection is considered to have worked satisfactorily.

**Figure 5:**
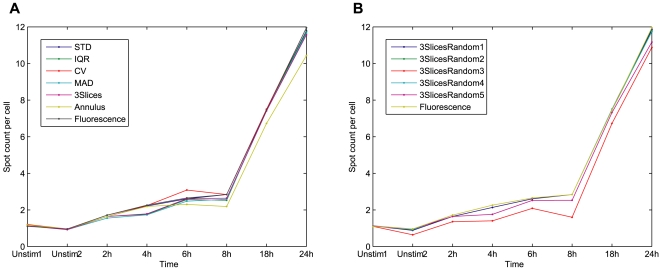
Spot enumeration, average number of spots per cell. Spots detected from fluorescence channel, and distributed among the cells based on different whole cell segmentation methods. (A) Spot counts per cell, cells detected from the bright field projections versus cells detected with the fluorescence reference. (B) Spot counts per cell, cells detected with standard deviation projections for five randomly selected slice triples. Only the Annulus method and 3SlicesRandom3 stand out as inferior to the others.

The results for this experiment are given in Fig. 5, where 5A shows the average spot counts per cell in each image for the different projection techniques, and in 5B the spot per cell enumeration is presented for the standard deviation projections of sets 3SlicesRandom1 to 5. With all the projection methods the spot count per cell increases over time, as previously reported in the literature [17].

**Figure 6:**
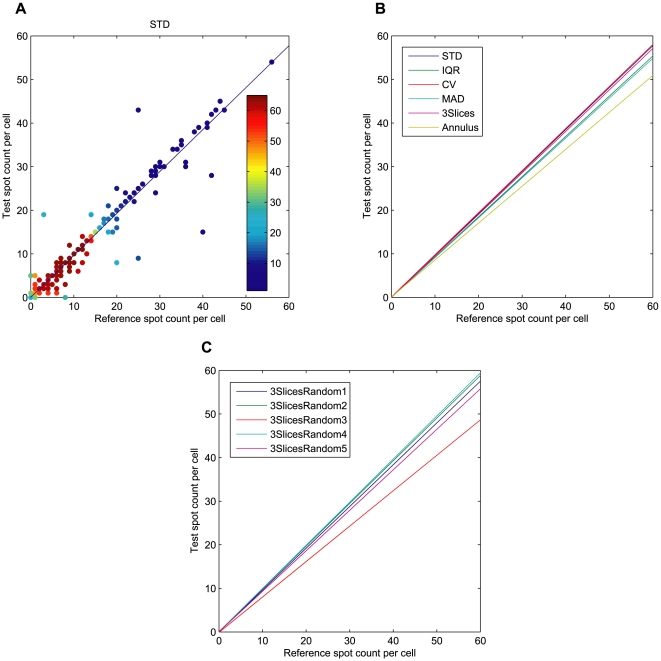
Cell by cell spot enumeration. Spots detected from fluorescence channel, and distributed among the cells based on different whole cell segmentation methods. (A) Each data point represents the number of spots in one cell, with cell area detected with standard deviation projection compared to cell area detection using fluorescence. The color indicates the number of overlapping data points. (B) Regression curves of spot counts cell by cell, with cell detection by each of the projection methods, the 3Slices method and the Annulus method against fluorescent ground truth. (C) Regression results of spot counts cell by cell, with cell detection of by the standard deviation projections for five randomly selected slice triples against fluorescence (sets 3SlicesRandom1–5).

Since each spot was assigned to a specific cell, we also compared the spot per cell counts for each individual cell for further validation. Fig. 6A shows a scatter plot and a regression curve obtained with linear least squares regression [18] of spot counts per each individual cell, for ground truth fluorescence against the STD projection. Overlapping data points are indicated with different colors. For clarity, in Fig. 6B only the regression lines are given for all the projections, with all the scatter plots available in the supplementary pages. Similarly to the previous plots, Fig. 6C illustrates the regression results for 3SlicesRandom1 to 5 against the ground truth fluorescence, with all the scatter plots again available as a supplement. The results of the spot-per-cell analysis are summarized in Table 2 listing the spot count slopes and biases for the different methods against ground truth. All the regression results except Annulus and the STD projection of 3SlicesRandom3 show a near perfect match between cell-by-cell spot counts by projections and fluorescence segmentation.

**Table 2:** Slopes and biases of spot per cell counts for all methods.

Segmentation method	Bias	Slope
STD	0.0524	0.9616
IQR	0.0130	0.9228
CV	0.1131	0.9656
MAD	−0.0135	0.9143
3Slices	−0.0068	0.9520
Annulus	0.1088	0.8467
3SlicesRandom1	0.0358	0.9584
3SlicesRandom2	0.0871	0.9797
3SlicesRandom3	−0.1181	0.8130
3SlicesRandom4	0.0773	0.9894
3SlicesRandom5	0.0256	0.9293

Spots are detected from fluorescence channel, but distributed among individual cells by whole cell detection based on the different methods. All methods except Annulus and 3SlicesRandom3 resulted in a near perfect match.

## Discussion

We have presented and evaluated different 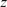 -projection methods for contrast enhancement in bright field image stacks, and shown that the projection approach can replace whole cell fluorescent staining for our set of macrophage images. In single cell detection and segmentation, our method has several advantages over the previously presented bright field based techniques. Firstly, the projection images can be directly used for whole cell segmentation in the freeware CellProfiler software or other tools. Secondly, among the different projection methods tested, the standard deviation projection is computationally very light and trivial to implement, requires no parameters to be set, and still offers excellent segmentation performance. Thirdly, we have successfully applied the whole cell detection method to macrophages, a cell type of high morphological complexity with various protrusions and low contrast. Fourthly, the segmentation results with randomly selected 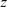 -slices suggest that precise focusing is not critical. And finally, background intensity variations have no effect on the resulting projection images. The drawback of our approach is the need for taking three images instead of one, requiring a rather fast stage in live cell imaging to acquire the images without cell movement, and currently the segmentation results include outliers resulting from erroneous whole cell detection. Space requirements, on the other hand, are not increased since only the projection images must be stored for analysis.

Further studies are needed for assessing generality of the projection approach. We only used images of one cell type, with low contrast all around the cells, without clearly visible cell borders. Halo effects, present in bright field images of many other cell types, for example yeast, might be emphasized erroneously in the projections. Furthermore, it would be interesting to study the segmentation performance with various cell densities and different imaging setups, and to search for optimal conditions for the imaging and subsequent analysis. Many different approaches could also be tested for preprocessing; in this work the standard Gaussian filter was found adequate, but no rigorous parameter optimization or method comparisons were performed.

To fully automate the bright field cell segmentation, the markers for each cell need to be located without fluorescent nuclei, but to the best of our knowledge, there are no robust bright field based methods presented in the literature. The markers could also be set manually, but especially in high throughput studies a manual approach is not realistic. In certain studies where the cells have a very distinctive shape, such us bacteria or yeast cells, the object separation could be done based on cell shape, removing the need for a nuclear marker and thus, the need for fluorescence altogether.

Bright field images are not the only stacks where the standard deviation or other projections should be studied in more detail. In fluorescence microscopy, the studied phenomenon is often visible as subcellular spots, the intensities varying according to the 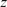 -levels. This suggests that the spots may be better visible in the standard deviation projections as compared to the methods commonly used, such as mean and maximum projections. The projection approach is also not limited to cellular objects, and any nearly transparent targets should benefit from the increased contrast without the need for any special optics.
